# Synthesis of C-coordinated O-carboxymethyl chitosan metal complexes and evaluation of their antifungal activity

**DOI:** 10.1038/s41598-018-23283-9

**Published:** 2018-03-19

**Authors:** Weixiang Liu, Yukun Qin, Song Liu, Ronge Xing, Huahua Yu, Xiaolin Chen, Kecheng Li, Pengcheng Li

**Affiliations:** 1 Key Laboratory of Experimental Marine Biology, Institute of Oceanology, Chinese Academy of Sciences, Qingdao, 266071 China; 2 Laboratory of Marine Drugs and Bioproducts of Qingdao National Laboratory for Marine Science and Technology, Qingdao, China; 3 University of Chinese Academy of Sciences, Beijing, 100049 China

## Abstract

Based on a condensation reaction, a chitosan-derivative-bearing amino pyridine group was prepared and subsequently followed by coordination with cupric ions, zinc ions and nickel ions to synthesize chitosan metal complexes. The calculations using the density functional theory (DFT) show that the copper ions and nickel ions underwent dsp^2^ hybridization, the zinc ions underwent sp^3^ hybridization, and they all formed a coordination bond with the carbon atom in the p-π conjugate group. The antifungal properties of O-CSPX-M against *Phytophthora capsici (P. capsici)*, *Verticillium alboatrum (V. alboatrum)*, *Botrytis cinerea (B. cinerea)* and *Rhizoctonia solani (R. solani)* were also assayed. Apparently, chitosan metal complexes showed enhanced antifungal activity against four fungi at the tested concentrations compared to that of chitosan. It was shown that Cu complexes can inhibit the growth of *P. capsici* 100%, and Ni complexes can inhibit the growth of *B. cinerea* 77.1% at a concentration of 0.4 mg/mL and 0.2 mg/mL, respectively. The pot experiment also verified the result. In addition, the phytotoxicity experiment showed that O-CSPX-M had no obvious toxicity on wheat leaves. This kind of complexes may represent as an attractive direction for chemical modifications of metal fungicides.

## Introduction

Organic metal complexes are an important branch of organic chemistry research. Many organic ligands exhibit excellent activity after their coordination with metal ions. Early in 1817, the first organometallic compound, Zeise salt (KC_2_H_4_PtCl_3_), was discovered. After that, the synthesis and application of organometallic compounds have been extensively studied, and considerable progress has been made. For example, oxaliplatin and nedaplatin have been widely used as antitumour drugs, and Ziegler-Natta catalysts greatly simplify the conditions for olefin polymerization. In recent years, organometallic complexes have been widely applied in many new fields, such as energetic materials^1^, photosensitizers^2^ and fungicides^3^.

Due to their outstanding biological activity, metal complexes have attracted increasing attention in agriculture. As early as 1761, the use of copper sulphate to sterilize seeds was recorded in agriculture. Up to the present, metal fungicides such as Bordeaux mixture have been used for over 100 years. Metal fungicides have the advantages of a wide spectrum of sterilization, non-resistance, low cost and non-volatilization^4,5^. However, traditional metal fungicides also have many disadvantages^6^. Inorganic metal fungicides, such as Bordeaux mixture, need to be used immediately and are not readily stored. Improper use can also cause phytotoxicity and mite outbreaks^7^. Organic metal fungicides, such as fosetyl-aluminium, have appeared in recent years and can be stored and transported, but many of them are still poisonous to the leaves of plants^6^. Therefore, in order to reduce the effect of traditional metal fungicides on crop growth, it is essential to develop novel organic metal fungicides with low toxicity, and low metal content.

Chitosan, a kind of renewable, abundant natural polysaccharide, consists primarily of 2-amino-2-deoxy-glucopyranose units linked by a β-(1–4) linkage^8–10^. It is readily obtained from chitin, which is one of the most abundant natural polysaccharides. It has been well-studied and sufficiently reported, earning the attention of scientific research^11,12^. However, because of its poor water solubility, chitosan usually needs to be modified. Therefore, the hydrophilic group, such as carboxymethyl, was introduced into the chitosan to improve its water solubility^13,14^.

Schiff base is a type of organic compound with good biological activity and can be coordinated with a large number of metal ions to form complexes^15–17^. Hence, the chitosan Schiff base metal complex with novel characteristic may have significant antifungal activity.

*Phytophthora capsici*, *Verticillium alboatrum, Botrytis cinerea* and *Rhizoctonia solani* are plant pathogens. They can cause diseases to a variety of vegetables, fruits and crops. The control of these threatened fungi is necessary. Therefore, the antifungal properties of metal complexes against these four kinds of fungi were evaluated.

## Results

### Chemical synthesis

**Figure 1:**
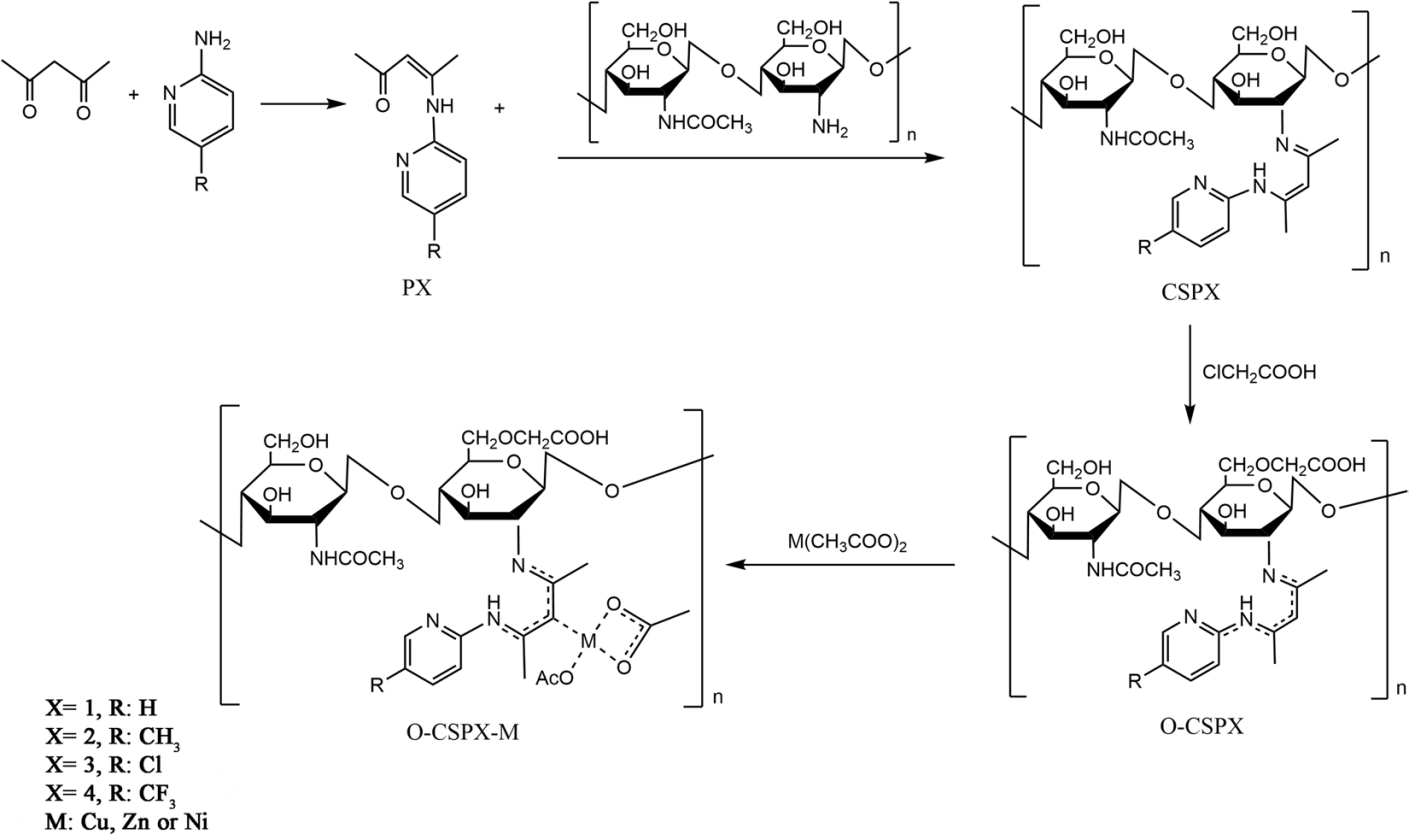
Synthetic route of O-carboxymethyl chitosan metal complexes (O-CSPX-M).

As shown in Fig. 1, an O-carboxymethyl chitosan metal complex was synthesized by four steps of reaction. First, we synthesized Schiff base ligands (PX), and then prepared the chitosan Schiff base (CSPX) by the same mechanism. Next, the water-soluble chitosan Schiff base (O-CSPX) was prepared by oxidizing the hydroxyl group of chitosan with chloroacetic acid. Finally, the metal complex (O-CSPX-M) was obtained through the reaction of Schiff base and metal acetate. The aforementioned chitosan derivatives were characterized by FT-IR, ^1^H NMR, ^13^C NMR, elemental analysis and XRD, and then proofed using Gaussian09.

### FT-IR

**Figure 2:**
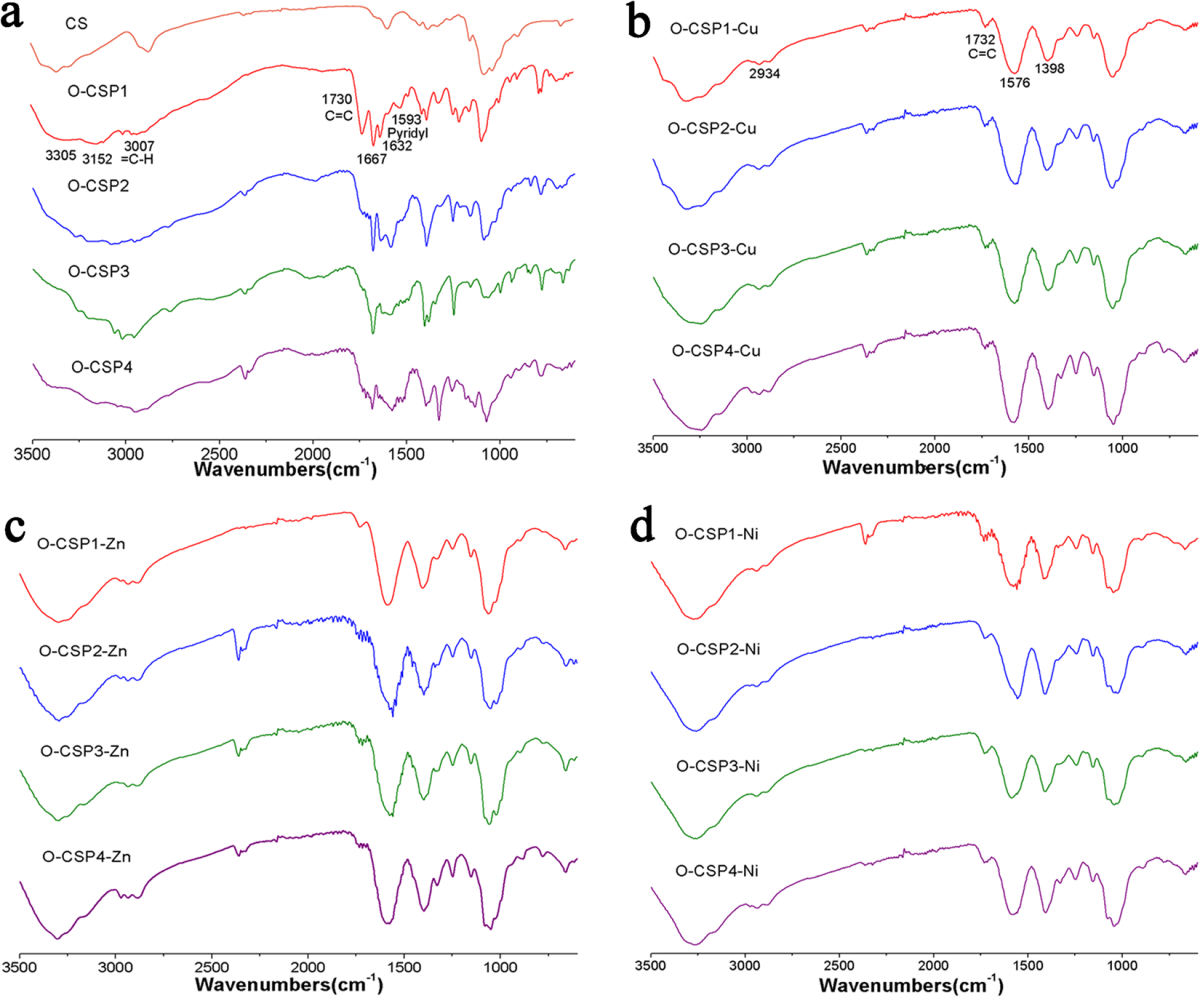
FT-IR spectra of chitosan (CS), O-carboxymethyl chitosan Schiff bases (O-CSPX) and O-carboxymethyl chitosan metal complexes (O-CSPX-M).

Figure 2 shows the FT-IR spectra of chitosan and chitosan derivatives. The broad band ranging from 3000 cm^−1^ to 3500 cm^−1^ of CS could be considered as -OH and -NH. The characteristic absorbance band of =C-H was observed at 3007 cm^−1^. The NH_2_ bending vibrations was shown at 1593 cm^−1^. In the spectrum of O-CSPX, an extra peak appeared at 1667 cm^−1^, which is the absorption peak of the stretching vibrations of C=O. The absorption peak of pyridyl appeared at 1525 cm^−1^, and the C=C was observed at 1730 cm^−1^. The band at 1409 cm^−1^ and the band at 1632 cm ^−1^, respectively, display the existence of C-N and C=N. The absorbance bands at 3305 cm^−1^ and 1209 cm^−1^ were attributed to the -OH group in the ring system.

As shown in Fig. 2, the C=C vibrations was observed at 1732 cm^−1^ in the spectrum of O-CSP1-Cu. Due to the coordination of the Cu(II) ions with the p-π conjugate group, the C=C vibrations of metal complexes shifted to a higher wave number in comparison of O-CSPX^18,19^. One additional signal was observed at 1397 cm^−1^ and was attributed to the carbonyl groups of CH_3_COO^−^.

### ^1^H NMR and elemental analysis

**Figure 3:**
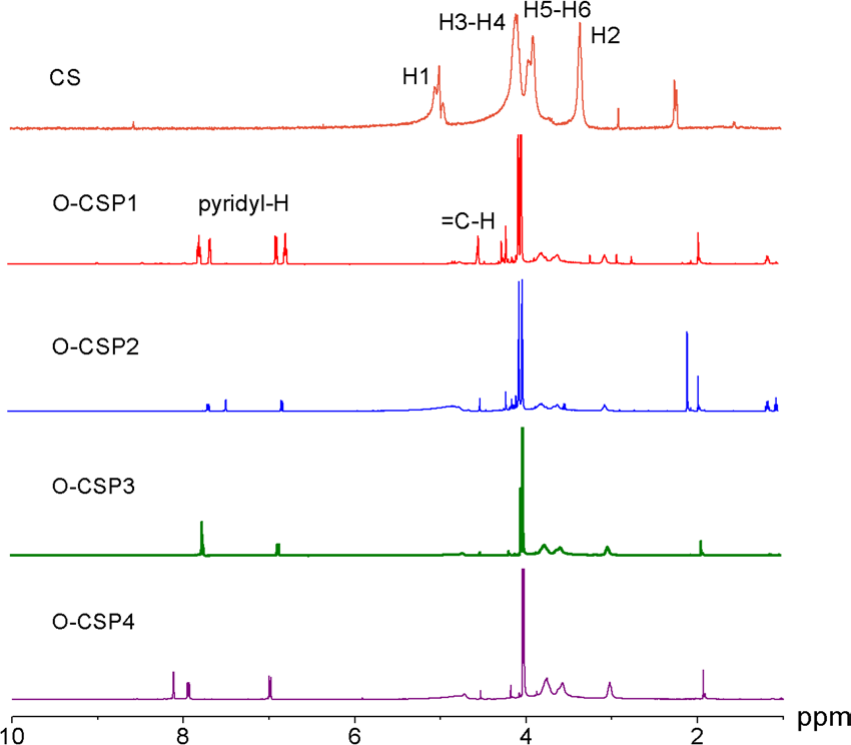
^1^H NMR spectra of O-carboxymethyl chitosan Schiff bases (O-CSPX).

The ^1^H NMR spectra of the CS and O-CSPX are shown in Fig. 3. The signals of CS were consistent with the previous observation^20,21^: (1) δ = 3.34 ppm: H2; (2) δ = 3.89–3.95 ppm: H5, H6; (3) δ = 4.07–4.09 ppm: H3, H4; (4) δ = 4.98 ppm: H1; (5) δ = 4.50 ppm: = C-H; (6) δ = 6.75, 6.87, 7.63, 7.76 ppm: pyridyl-H.

The degree of deacetylation (DD) of CS was calculated using the equation given below^22^.

1 $${\rm{DD}}=[1-\frac{({\rm{C}}{\rm{/}}{\rm{N}}-5.145)}{6.681-5.145}]\times 100$$

The degree of substitution (DS) of O-CSPX-M was estimated employing the following formula^23,24^:

2 $${\rm{DS}}=\frac{{(\alpha {\rm{C}}{\rm{/}}{\rm{N}})}_{{\rm{m}}}-{({\rm{C}}{\rm{/}}{\rm{N}})}_{{\rm{o}}}}{n}$$

The (C/N) _m_ is the C/N of the O-CSPX, (C/N)_o_ is the C/N of the CS, and *a* and *n* are the amount of nitrogen and carbon introduced after modification, respectively.

The results of elemental analysis are shown in Supplementary Table S1. The DS of O-CSP4-Cu, O-CSP4-Zn and O-CSP4-Ni was 76.54%, 74.19% and 73.04%, respectively, relatively higher than that of the others, whose values were approximately 70%.

### ^13^C NMR

**Figure 4:**
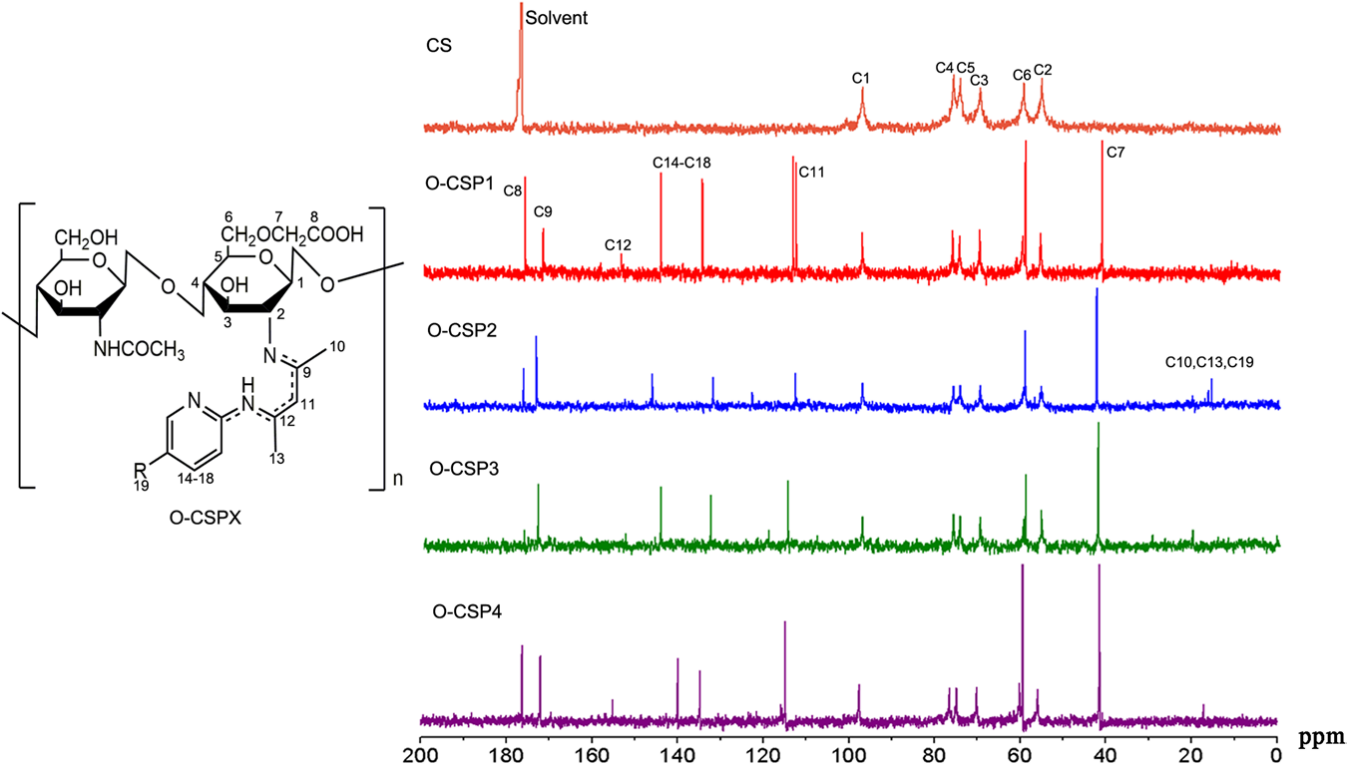
^13^C NMR spectra of O-carboxymethyl chitosan Schiff bases (O-CSPX).

The ^13^C NMR spectra of the CS and O-CSPX are shown in Fig. 4. In CS, the signals can be interpreted as δ = 97.4 ppm (C1), 76.3 ppm (C4), 74.6 ppm (C5), 69.9 ppm (C3), 59.8 ppm (C6), and 55.6 ppm (C2), this is in accordance with the previous reports. In O-CSPX, there are some new peaks: (1) δ = 42.6 ppm: C7; (2) δ = 176.6 ppm: C8; (3) δ = 173.5 ppm: C9; (4) δ = 165–113.6 ppm: C11; (5) δ = 153.7 ppm: C12; (6) δ = 134, 144 ppm: pyridyl-C.

### XRD

**Figure 5:**
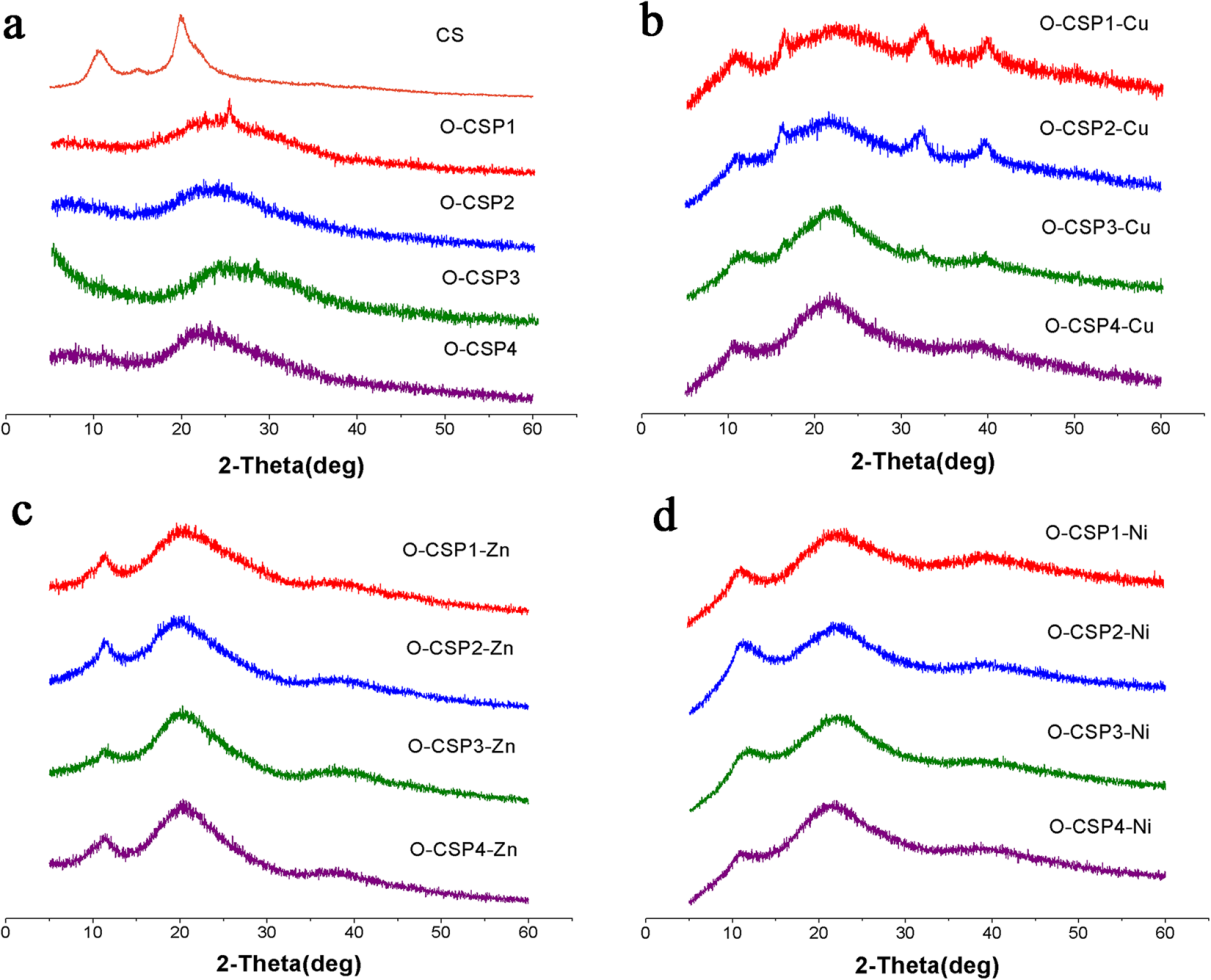
XRD curves of chitosan (CS), O-carboxymethyl chitosan Schiff bases (O-CSPX) and O-carboxymethyl chitosan metal complexes (O-CSPX-M).

The water solubility of chitosan is poor. This is due to the existence of a large number of hydrogen bonds in the molecule, which restricts the free rotation of the units in each group. From Fig. 5, the crystal reflection angle of CS is observed at 11° and 20°, which is consistent with the previous literature. In the spectra of the O-CSPX, the wide and strong peak at 8° and 22° could be considered as the crystal reflection angle. The introduction of Schiff base on chitosan leads to changes in peak shape, but also improves the water solubility. In the spectra of the O-CSPX-M, the crystal reflection angle was observed at 11°, 23° and 39°. The results show that the crystal structure of O-CSPX was further damaged by the coupling effect, and the chemical reaction changed its crystal structure.

### Antifungal assay (*in vitro*)

In this paper, chitosan and its derivatives were dissolved in 0.5% (v/v) acetic acid at an original concentration of 8% (w/v). Because acetic acid can enhance the antifungal action, in order to exclude the effect of acetic acid on the experimental results, we determined the inhibitory effect of acetic acid against four fungi under different concentrations^25^. The results are shown that the acetic acid aqueous solution would not enhance the antifungal efficacy at concentrations lower than 0.125 mg/mL (Figure S3).

The antifungal activities of the metal complexes against *P. capsici*, *V. alboatrum*, *B. cinerea* and *R. solani* are shown in Table 1. We used chitosan and Cuproxat as the control group. Cuproxat is a widely used commercially available copper fungicide. In general, the antifungal effect of all metal complexes is better than that of chitosan, especially to *P. capsici* and *B. cinerea*. The inhibition rate of O-CSPX-M against *R. solani* ranged from 31.84% to 64.80%, and the antifungal activity of O-CSPX-Cu was better than the others. Moreover, its inhibitory effect was much better than that of Cuproxat at low concentrations.

**Table 1:** Antifungal activity of twelve types of metal complexes (O-CSPX-M) against *P. capsici, V. alboatrum, B. cinerea* and *R. solani*.

Sample	Concentration(mg/mL)	Inhibitory index (%)	*V. alboatrum*	*B. cinerea*	*R. solani*
		*P. capsici*			
Chitosan	0.1	7.61 ± 3.52^bc^	-5.59 ± 1.08^a^	32.34 ± 2.28^abcdef^	5.03 ± 1.94^a^
	0.2	16.24 ± 10.66^bcdef^	-4.97 ± 3.88^ab^	26.87 ± 1.49^abcd^	11.73 ± 2.56^a^
	0.4	37.06 ± 11.83^lmn^	3.11 ± 3.23^defg^	38.81 ± 0^cdefghi^	24.02 ± 4.22^b^
O-CSP1-Cu	0.1	−5.58 ± 0.88^a^	3.73 ± 5.69^gh^	31.24 ± 3.9^abcde^	53.63 ± 5.12^ghijklmn^
	0.2	55.33 ± 9.91°	7.45 ± 2.85^gh^	26.87 ± 0^abcd^	58.1 ± 2.9^jklmn^
	0.4	100 ± 0^q^	55.28 ± 1.86 ^m^	21.39 ± 3.11^ab^	55.87 ± 0.97^hijklmn^
O-CSP2-Cu	0.1	−4.06 ± 1.76^a^	15.53 ± 1.08^jk^	23.88 ± 2.59^abc^	60.34 ± 2.56^klmn^
	0.2	55.33 ± 6.34°	16.15 ± 1.86^k^	29.85 ± 5.38^abcde^	62.01 ± 5.39^lmn^
	0.4	100 ± 0^q^	62.11 ± 1.08^n^	35.82 ± 7.76^abcdefgh^	63.69 ± 5.12^mn^
O-CSP3-Cu	0.1	37.06 ± 3.52^n^	10.56 ± 4.93^hi^	23.38 ± 6.03^abc^	58.1 ± 1.68^jklmn^
	0.2	76.14 ± 0.88^p^	13.04 ± 1.08^ijk^	31.34 ± 7.46^abcde^	55.87 ± 2.56^hijklmn^
	0.4	100 ± 0^q^	64.6 ± 1.86^n^	40.8 ± 6.73^defghij^	63.69 ± 4.84^mn^
O-CSP4-Cu	0.1	36.55 ± 2.33^mn^	7.45 ± 1.08^gh^	30.35 ± 12.69^abcde^	50.84 ± 3.49^efghijkl^
	0.2	78.68 ± 1.52^p^	15.53 ± 3.88^jk^	37.31 ± 7.76^bcdefgh^	57.54 ± 0.97^ijklmn^
	0.4	100 ± 0^q^	55.9 ± 3.88 ^m^	34.33 ± 5.17^abcdefgh^	64.8 ± 1.68^n^
O-CSP1-Zn	0.1	26.9 ± 4.03^ijklmn^	1.86 ± 2.85^cdef^	29.85 ± 9.08^abcde^	46.93 ± 2.56^defghij^
	0.2	31.98 ± 7.03^klmn^	1.24 ± 1.86^cde^	32.84 ± 11.66^abcdefg^	47.49 ± 3.49^defghij^
	0.4	31.98 ± 3.83^klmn^	3.11 ± 3.73^defg^	24.38 ± 1.72^abc^	47.49 ± 0.97^defghij^
O-CSP2-Zn	0.1	23.35 ± 0.88^fghijkl^	6.83 ± 1.86^fgh^	21.89 ± 6.03^ab^	51.4 ± 4.43^fghijkl^
	0.2	29.95 ± 0^jklmn^	3.11 ± 3.23^defg^	25.87 ± 6.03^abcd^	48.04 ± 2.9^defghij^
	0.4	25.38 ± 1.52^ghijkl^	11.18 ± 2.15^hij^	28.36 ± 9.32^abcde^	51.96 ± 3.87^ghijklm^
O-CSP3-Zn	0.1	21.83 ± 0.88^fghijk^	0.62 ± 1.08^cde^	21.89 ± 1.72^ab^	45.25 ± 4.22^defgh^
	0.2	26.4 ± 2.33^hijklm^	4.97 ± 3.23^efg^	43.28 ± 11.85^efghijk^	43.58 ± 7.92^defg^
	0.4	29.44 ± 7.51^jklmn^	3.73 ± 2.85^defg^	39.3 ± 4.56^cdefghi^	48.6 ± 7.56^defghijk^
O-CSP4-Zn	0.1	18.27 ± 1.76^cdefghi^	-1.24 ± 1.08^abcd^	36.82 ± 3.76^abcdefgh^	31.84 ± 15.84^bc^
	0.2	15.74 ± 7.51^bcdefgh^	4.35 ± 2.15^efg^	21.89 ± 11.4^ab^	51.4 ± 2.9^fghijkl^
	0.4	20.3 ± 12.77^defghij^	10.56 ± 3.73^hi^	34.83 ± 7.05^abcdefgh^	46.93 ± 3.49^defghij^
O-CSP1-Ni	0.1	19.8 ± 12.31^defghij^	1.86 ± 2.85^cdef^	33.83 ± 8.75^abcdefg^	45.25 ± 4.84^defgh^
	0.2	20.81 ± 5.49^defghij^	3.11 ± 1.86^defg^	53.73 ± 11.66^ijklm^	45.81 ± 9.23^defghi^
	0.4	14.72 ± 13.19^bcdefg^	−1.24 ± 2.85^abcd^	63.68 ± 9.12 ^m^	55.87 ± 7.56^hijklmn^
O-CSP2-Ni	0.1	16.24 ± 8.48^bcdefghi^	0 ± 1.08^bcde^	43.78 ± 9^efghijk^	38.55 ± 6.35 ^cd^
	0.2	21.32 ± 6.15^efghij^	4.35 ± 1.08^efg^	50.25 ± 9.94^hijklm^	39.66 ± 3.35^cdef^
	0.4	26.4 ± 0.88^hijklm^	0 ± 2.15^bcde^	77.11 ± 6.03^n^	53.63 ± 11.16^ghijklmn^
O-CSP3-Ni	0.1	10.15 ± 3.05^bcd^	0.62 ± 2.85^cde^	47.76 ± 4.48^fghijkl^	46.93 ± 5.89^defghij^
	0.2	13.2 ± 1.52^bcdef^	0 ± 2.15^bcde^	43.78 ± 9.12^efghijk^	45.81 ± 8.27^defghi^
	0.4	10.66 ± 3.83^bcde^	3.11 ± 1.86^defg^	58.21 ± 15.58^klm^	45.25 ± 3.49^defgh^
O-CSP4-Ni	0.1	13.2 ± 2.64^bcdef^	−1.24 ± 4.69^abcd^	34.33 ± 10.45^abcdefgh^	46.93 ± 8.27^defghij^
	0.2	16.75 ± 4.65^bcdefghi^	0.62 ± 2.85^cde^	41.29 ± 8.62^defghij^	46.37 ± 3.35^defghij^
	0.4	20.81 ± 4.03^defghij^	−3.11 ± 3.88^abc^	56.22 ± 17.23^jklm^	56.98 ± 13.65^hijklmn^
Cuproxat	0.1	6.6 ± 6.15^b^	9.94 ± 1.08^hi^	20.9 ± 1.49^a^	21.79 ± 12.8^b^
	0.2	100 ± 0^q^	14.29 ± 1.86^ijk^	48.76 ± 11.59^ghijklm^	39.11 ± 2.56^cde^
	0.4	100 ± 0^q^	32.3 ± 2.15 ^l^	59.7 ± 0 ^lm^	75.98 ± 2.56°

Three replicates of each test were carried out, and the values were expressed as percentages. Different letters indicate significant differences between groups (P < 0.05) according to variance analysis (ANOVA) using the SPSS software, and the means were compared by Duncan’s multiple comparison post-test.

As can be seen from Table 1, the modification of chitosan can significantly improve its antifungal activity. Among them, the copper complexes exhibited relatively high inhibitory effect against *P. capsici* and *V. alboatrum*, and the highest antifungal index was 100% and 64.6%, respectively. The inhibition effect of nickel complexes against *B. cinerea* was relatively high, and the inhibition rate of nickel complexes could be higher than 77.11% against *B. cinerea* at a concentration of 0.4 mg/mL. The zinc complex showed weaker antifungal activity relative to the other two complexes, but the antifungal index was still higher than that of highly deacetylated chitosan. This shows that the antifungal activity of chitosan derivatives can be strengthened by grafting metal ions. As seen in Table 1, there is no significant difference among O-CSP1-M, O-CSP2-M, O-CSP3-M and O-CSP4-M, which means the structural difference has no significant effect on the antifungal activity of O-CSPX-M. In addition, the inhibition indexes of some metal complexes are higher than that of Cuproxat at the same concentration.

The antifungal results show that the antifungal activity of O-CSPX-M is hardly affected by the types of pyridyl. Combined with our prior research^14^, this is because the substituent on the pyridine ring is far from the complexation site, and the influence on the dihedral angle in molecular was very small. Hence, a super-conjugated system was formed in O-CSPX-M, and the bond energy of the coordination bond was enhanced.

The bioassay results show that the O-carboxymethyl chitosan metal complexes can obviously inhibit the growth of fungi. They all show broad-spectrum antifungal activity. The antifungal activity of these metal complexes is attributed to their ability to trigger cell death when they are attached to negatively charged microbial cells causing irreversible damage to their cell membrane^26^. The results further confirm that metal ions grafted in the synthesized chitosan derivatives contribute a lot to the antifungal action.

### Protective and Curative Activity (*in vivo*)

The protective and curative activity of the chitosan derivatives are shown in Table 2. The disease indexes of pepper seedlings irrigated with chitosan derivatives were significantly lower than that of the negative control group irrigated with water. Moreover, the control efficacy increases with the increase of concentration. At the concentration of 0.8 mg/mL, the highest protective efficacy of O-CSPX-Cu was 85.56% and the highest curative efficacy was 74.41%, slightly higher than that of the positive control Cuproxat. In addition, the efficacy obtained by O-CSPX-Cu for protective activity was always higher than that for curative activity at the same concentration.

**Table 2:** Protective and curative activity of twelve types of metal complexes (O-CSPX-M) against *P. capsici*. Values are means of three pepper seedlings and from three independent experiments. Different letter indicated significant differences between groups (P < 0.05) according to variance analysis (ANOVA) using the SPSS software and means were compared by Duncan’s multiple comparison post-test.

Sample	Concentration(mg/mL)	Protective Activity		Curative Activity	
		Disease Index	Control Efficacy (%)	Disease Index	Control Efficacy (%)
O-CSP1-Cu	0.4	47.22 ± 4.81^bc^	36.48 ± 10.19^ab^	63.89 ± 12.73 ^cd^	26.26 ± 7.62^a^
	0.8	19.44 ± 20.97^a^	75.93 ± 25.05^c^	25.00 ± 14.43^ab^	71.38 ± 14.72^b^
O-CSP2-Cu	0.4	22.22 ± 12.73^a^	71.02 ± 16.00^c^	50.00 ± 8.33^bc^	42.09 ± 4.98^ab^
	0.8	11.11 ± 12.73^a^	85.56 ± 17.11^c^	22.22 ± 17.35^a^	74.41 ± 18.41^b^
O-CSP3-Cu	0.4	33.33 ± 14.43^ab^	55.28 ± 19.37^bc^	66.67 ± 14.43 ^cd^	23.23 ± 8.75^a^
	0.8	11.11 ± 12.73^a^	85.06 ± 16.48^c^	25.00 ± 16.67^ab^	70.71 ± 18.51^b^
O-CSP4-Cu	0.4	27.78 ± 4.81^ab^	63.06 ± 3.37^bc^	69.44 ± 4.81 ^cd^	18.18 ± 15.75^a^
	0.8	13.89 ± 12.73^a^	82.59 ± 15.57^c^	27.78 ± 20.97^ab^	67.68 ± 22.74^b^
Cuproxat	0.4	55.56 ± 12.73 ^cd^	24.54 ± 22.06^a^	61.11 ± 12.73 ^cd^	28.62 ± 14.72^a^
	0.8	16.67 ± 8.33^a^	77.22 ± 11.82^c^	27.78 ± 12.73^ab^	66.33 ± 19.49^b^
H_2_O	—	75.00 ± 8.33^d^	—	86.11 ± 9.62^d^	—

### Phytotoxicity assay

As is shown in Table 3, all of the three free metal ion solutions could significantly decrease the contents of total chlorophyll by 8.1–12.7% at 0.2 mg/mL. O-CSPX-Cu and O-CSPX-Ni could decrease Chl-a, Chl-b and total chlorophyll by 7.8–15.6%, −11.2–14.6% and 5.8–12.5%, respectively, at 0.2 mg/mL, lower than that of the free metal ion solutions. However, O-CSPX-Zn at 0.2 mg/mL could increase Chl-a, Chl-b and total chlorophyll by 1.3–9.1%, 2.2–21.8% and 6.4–7.2%, respectively. This is because chitosan and low concentrations of zinc ions both promote the growth of plants^27^. The Schiff base partially counteracts the growth inhibition effect of metal ions and reduces the damage caused by O-CSPX-M to plants.

**Table 3:** Size effects of O-CSPX-M on chlorophyll contents of wheat seedlings.

Sample	Concentration (mg/mL)	Chl-a (mg/g)	Chl-b (mg/g)	Chl(a + b) (mg/g)
O-CSP1-Cu	0.1	0.887 ± 0.059^defghij^	0.363 ± 0.013^cdefghi^	1.251 ± 0.058^def^
	0.2	0.825 ± 0.035^abcde^	0.314 ± 0.020^bcd^	1.139 ± 0.054^abc^
O-CSP2-Cu	0.1	0.956 ± 0.031^ijklm^	0.305 ± 0.025^abc^	1.261 ± 0.042^defgh^
	0.2	0.832 ± 0.031^abcdefg^	0.316 ± 0.017^bcd^	1.148 ± 0.047^abc^
O-CSP3-Cu	0.1	0.902 ± 0.104^efghijk^	0.355 ± 0.022^bcdefghi^	1.258 ± 0.084^defg^
	0.2	0.796 ± 0.040^abc^	0.397 ± 0.035^fghhijk^	1.193 ± 0.012^abcde^
O-CSP4-Cu	0.1	0.803 ± 0.067^abc^	0.411 ± 0.050^hijk^	1.214 ± 0.037^bcde^
	0.2	0.784 ± 0.006^ab^	0.391 ± 0.016^efghij^	1.175 ± 0.014^abcde^
O-CSP1-Zn	0.1	0.974 ± 0.037^klm^	0.365 ± 0.021^cdefghi^	1.339 ± 0.058^fghi^
	0.2	0.970 ± 0.048^klm^	0.378 ± 0.025^efghij^	1.348 ± 0.073^ghi^
O-CSP2-Zn	0.1	0.981 ± 0.042^klm^	0.368 ± 0.027^defghi^	1.349 ± 0.069^ghi^
	0.2	0.966 ± 0.054^jklm^	0.387 ± 0.026^efghij^	1.353 ± 0.078^hi^
O-CSP3-Zn	0.1	0.878 ± 0.039^ghijkl^	0.451 ± 0.021^k^	1.329 ± 0.046^fghi^
	0.2	0.922 ± 0.033^hijklm^	0.435 ± 0.050^jk^	1.356 ± 0.025^i^
O-CSP4-Zn	0.1	0.992 ± 0.026 ^lm^	0.365 ± 0.016^cdefghi^	1.356 ± 0.042^i^
	0.2	0.993 ± 0.055 ^m^	0.365 ± 0.075^cdefghi^	1.358 ± 0.036^i^
O-CSP1-Ni	0.1	0.878 ± 0.052^cdefghi^	0.359 ± 0.018^bcdefghi^	1.236 ± 0.069^cde^
	0.2	0.803 ± 0.026^abc^	0.305 ± 0.020^abc^	1.109 ± 0.045^a^
O-CSP2-Ni	0.1	0.818 ± 0.028^abcd^	0.402 ± 0.063^ghijk^	1.220 ± 0.041^cde^
	0.2	0.839 ± 0.019^abcdefg^	0.350 ± 0.006^bcdefgh^	1.190 ± 0.024^abcde^
O-CSP3-Ni	0.1	0.856 ± 0.044^bcdefgh^	0.357 ± 0.019^bcdefghi^	1.214 ± 0.064^bcde^
	0.2	0.768 ± 0.020^a^	0.353 ± 0.006^bcdefgh^	1.122 ± 0.025^ab^
O-CSP4-Ni	0.1	0.808 ± 0.038^abcd^	0.415 ± 0.030^ijk^	1.223 ± 0.028^cde^
	0.2	0.830 ± 0.032^abcdef^	0.334 ± 0.011^bcde^	1.164 ± 0.043^abccd^
Cu(OAc)_2_	0.1	0.950 ± 0.026^ijklm^	0.257 ± 0.022^a^	1.207 ± 0.047^bcde^
	0.2	0.805 ± 0.040^abcd^	0.301 ± 0.037^ab^	1.106 ± 0.049^a^
Zn(OAc)_2_	0.1	0.855 ± 0.012^bcdefgh^	0.346 ± 0.039^bcdefg^	1.200 ± 0.043^abcde^
	0.2	0.819 ± 0.024^abcd^	0.368 ± 0.026^defghi^	1.188 ± 0.020^abcde^
Ni(OAc)_2_	0.1	0.808 ± 0.034^abcd^	0.403 ± 0.049^ghijk^	1.211 ± 0.016^bcde^
	0.2	0.786 ± 0.044^ab^	0.379 ± 0.018^efghij^	1.165 ± 0.026^abcd^
Cuproxat	0.1	0.813 ± 0.016^abcd^	0.416 ± 0.014^ijk^	1.229 ± 0.029^cde^
	0.2	0.840 ± 0.048^abcdefg^	0.336 ± 0.021^bcdef^	1.176 ± 0.069^abcde^
H_2_O		0.910 ± 0.054^fghijk^	0.357 ± 0.020^bcdefghi^	1.267 ± 0.074^efghi^

Values are the mean ± SD of three replicates. Different letters indicate significant differences between groups (P < 0.05) according to variance analysis (ANOVA) using the SPSS software, and the means were compared by Duncan’s multiple comparison post-test.

## Discussion

Metal fungicides are widely used because of their inhibitory effects on many plant pathogenic fungi^28–30^. However, improper use of metal fungicides can affect crop growth. Based on past soil damage caused by pesticide abuse, the development of new organic chemicals with high-efficiency, low-toxicity and low metal content is imminent. In this study, we have proposed a straightforward synthetic route to novel metal complexes coordinated with chitosan Schiff bases. The antifungal effect of the metal complexes against *P. capsici*, *V. alboatrum*, *B. cinerea* and *R. solani* was evaluated. The results showed that all twelve metal complexes could effectively inhibit the selected fungi. The inhibition rate of some metal complexes is better than that of commercial pesticide Cuproxat at the same dose. Moreover, a phytotoxicity assay of O-CSPX-M was also conducted, and unlike the traditional metal fungicides, the twelve metal complexes were not significantly toxic to the leaves of wheat. O-CSPX-Zn can even increase chlorophyll content in wheat leaves at low concentration. This is mainly because chitosan itself promotes plant growth and counteracts the phytotoxicity of metal ions.

For metal complexes, the hybrid forms and ligand fashion of metal ions are an important part of their structural analysis. However, many previous reports have focused on the activities, and few have paid attention to the ligand fashion^31,32^. This is because the inner orbit of transition metal ions after coordination is filled with electrons, displayed as paramagnetism. Therefore, they cannot be analysed using NMR. Thus, in this study, we used density functional theory to calculate the specific configuration of the complexes. The calculations show that the copper ions and nickel ions underwent dsp2 hybridization, the zinc ions underwent sp3 hybridization, and they all formed a coordination bond with the carbon atom in the p-π conjugate group due to steric hindrance. The characterization of metal complexes provides an attractive direction for chemical modifications.

In conclusion, this study provides insights into the synthesis of metal fungicides from marine organisms. Compared to the metal fungicides used, the chitosan metal complexes had better antifungal activity, lower phytotoxicity and lower metal content. This kind of complexes may represent as an attractive direction for chemical modifications of metal fungicides.

## Materials and Methods

### Materials

Chitosan (degree of Deacetylation 81%, average-molecular weight 1300 kDa), was purchased from Qingdao Yun Zhou Biochemistry Co., Ltd. 2-Aminopyridine, 2-Amino-5-methylpyridine, 2-Amino-5-chloropyridine and 2-Amino-5-(trifluoromethyl)pyridine were purchased from Shanghai Aladdin Bio-Chem Technology Co., Ltd. The other reagents are all of analytical grade and used without further purification. Four crop-threatening pathogenic fungi (*P. capsici*, *V. alboatrum*, *B. cinerea* and *R. solani*) were provided by Qingdao Academy of Agricultural Sciences.

### Analytical methods

FT-IR spectra were recorded on a Thermo Scientific Nicolet iS10 FT-IR spectrometer with KBr disks. ^1^H NMR and ^13^C NMR spectra were measured with a JEOL JNM-ECP600 spectrometer. The elemental analysis (C, H, N, Cu, Zn and Ni) was performed using a Vario EL-III elemental analyser. All geometries were fully optimized at the B3LYP/6–31 + G (d, p) level and the B3LYP/Lanl2DZ level of theory with the Gaussian 09 suite of programs.

### Synthesis of Schiff base ligands (PX)

First, four types of aminopyridine (0.05 mol) and acetylacetone (5 g, 0.05 mol) were added to ethanol (150 mL) and heated at 80 °C for 12 h. The solution was concentrated under reduced pressure. Then the orange crystal solid was washed with cold ethanol for three times, drying in the oven at 50 °C for 12 h^14^ (yields of 65.4%, 56.3%, 58.5% and 69.2%).

### Preparation of highly deacetylated chitosan (CS)

Highly deacetylated chitosan was prepared as previously described in the literature^14^.

### Synthesis of O-carboxymethyl chitosan Schiff base (O-CSPX)

First, CS (1.5 g) was added to 75 mL distilled water in a three neck flask, 60 mL ethanol and 1.5 mL of acetic acid were added, and the solution was stirred for 1 h. Then, the PX (two-fold that of CS) was dissolved in 50 mL of ethanol, and added dropwise into the three neck flask. After stirring for 15 h at 60 °C^14^, 7.5 g of monochloroacetic acid in 20 mL of ethanol was added dropwise. The mixture was heated at 60 °C for 6 h. Then, the mixture was precipitated by the addition of excess ethanol and the precipitate was filtered. The products were washed with ethanol and dried at 60 °C for 6 h (yields of 87.7%, 82.4%, 86.8% and 91.4%).

### Synthesis of O-carboxymethyl chitosan metal complexes (O-CSPX-M)

A solution of 1.0 g O-CSPX and Cu (CH_3_COO) _2_·H_2_O (1.1 g), Zn (CH_3_COO) _2_·2H_2_O (1.2 g) or Ni (CH_3_COO) _2_·4H_2_O (1.3 g) in 100 mL water was stirred for 6 h at 60 °C. The solution was precipitated with excess ethanol and centrifuged. The metal complexes were obtained after drying at 50 °C for 6 h. The product yield was 67.1% for O-CSP1-Cu, 66.2% for O-CSP2-Cu, 65.5% for O-CSP3-Cu, 50.1% for O-CSP4-Cu, 65.5% for O-CSP1-Zn, 75.2% for O-CSP2-Zn, 56.1% for O-CSP3-Zn, 54.7% for O-CSP4-Zn, 57.1% for O-CSP1-Ni, 49.9% for O-CSP2-Ni, 56.7% for O-CSP3-Ni and 47.4% for O-CSP4-Ni.

### Antifungal assay

Antifungal assays were performed by following the plate growth rate method^33^. The antifungal activity was evaluated *in vitro* against *P. capsici*, *V. alboatrum*, *B. cinerea* and *R. solani*. The tested concentrations were 0.1 mg/mL, 0.2 mg/mL and 0.4 mg/mL, respectively.

Each experiment was performed three times, and the data were shown with means ± SD. The Duncan’s multiple comparison test was used to evaluate the differences in antifungal index in the antifungal tests. Results with P < 0.05 were considered statistically significant

### Protective and Curative Activity (*in vivo*)

The protective and curative activity of O-carboxymethyl chitosan copper complexes against *P. capsici* was tested according to a previous study with some modifications^34^. For protective activity, pepper seedlings with at least ten true leaves were irrigated with 5 mL of test reagents. The tested concentrations were 0.40 mg/mL and 0.80 mg/mL, respectively. After 24 h, pepper seedlings were irrigated with 1 mL of spore suspension (3.5 × 10^3^). For curative activity, pepper seedlings were irrigated with the treatment as above at 24 h after irrigated with spore suspension (3.5 × 10^3^). Then the irrigated plants were kept at 25 °C with 85% humidity for 7 days. The disease index and control efficacy were calculated^34^. Three pepper seedlings per pot and three pots per concentration were used.

### Phytotoxicity assay

The present study was conducted with wheat (*Triticum aestivum* L. Jimai 22) seeds. After being sterilized, they were transferred to a Petri dish with moist gauze for germination at 25 °C for 24 h in the dark. Then, 2,736 germinated seeds were individually transplanted to 57 Petri dishes with nylon mesh, and each Petri dish contained 48 seeds. Wheat seedlings were cultivated in a growth incubator with a light intensity of 800 mol m^−2^ s^−1^, a day/night cycle of 14 h/10 h at 25 °C/15 °C, respectively, and the relative humidity was controlled at 70%. When the second functional leaf of wheat seedlings was fully expanded, the experiments were randomly divided into 33 groups, including a negative control group (sprayed with distilled water), 6 free metal ion groups (sprayed with 0.1 mg/mL and 0.2 mg/mL of free metal ion solution), 2 positive control groups (sprayed with 0.1 mg/mL and 0.2 mg/mL of commercial pesticide Cuproxat solution) and 24 metal complex groups (sprayed with 0.1 mg/mL and 0.2 mg/mL of O-CSPX-M solution), and each group had three replicates. The volume of distilled water, free metal solutions or O-CSPX-M solutions sprayed on each sample was 45 mL. Additionally, the wheat seedlings were cultured with Hoagland solution, and the solution was renewed every other day.

In this test, we used chlorophyll contents to represent phytotoxicity. The content of chlorophyll was determined using the method of Sedmak and Grossberg^35^. After 1 d of O-CSPX-M treatments, chlorophyll was extracted with 95% ethanol. Chlorophyll a (Chl-a), chlorophyll b (Chl-b) and total chlorophyll (a + b) content were determined using a spectrophotometer at 665 nm and 649 nm. All the processes, from extraction to spectral measurement, were performed in dim light to avoid the degradation of the chlorophyll in the samples.

## Electronic supplementary material

Supplementary Information
