# Supplementary material for: Synthesis of C-coordinated O-carboxymethyl chitosan metal complexes and evaluation of their antifungal activity
Source: Sci Rep. 2018 Mar 19;8:4845. doi: 10.1038/s41598-018-23283-9 (PMC5859048; doi:10.1038/s41598-018-23283-9)
Supplement: Supplementary file 1 — Supplementary Information [file 41598_2018_23283_MOESM1_ESM.docx]

**Synthesis of** **C-****coordinated O-carboxymethyl chitosan metal complexes and evaluation of their antifungal activity**

Weixiang Liu^1, 2, 3^, Yukun Qin^1, 2, *^, Song Liu^1, 2^, Ronge Xing^1, 2^, Huahua Yu^1, 2^, Xiaolin Chen^1, 2^, Kecheng Li^1, 2^, Pengcheng Li^1, 2, *^

1 Key Laboratory of Experimental Marine Biology, Institute of Oceanology, Chinese Academy of Sciences, Qingdao 266071, China

2 Laboratory of Marine Drugs and Bioproducts of Qingdao National Laboratory for Marine Science and Technology

3 University of Chinese Academy of Sciences, Beijing 100049, China

* Author to whom correspondence should be addressed: E-Mail: pcli@qdio.ac.cn (P.-C.L.);

ykqin@qdio.ac.cn (Y. -K.Q).

**Supplementary data**

**Results**

**Gaussian 09**

We optimize the structures of O-CMPX and O-CMPX-M at the B3LYP/6-31+G (d, p) level and the B3LYP/Lanl2DZ level of theory using the Gaussian 09 suite of programs. The lowest energy forms of O-CMPX are shown in Figure S1. As shown in the figure, because there are no large groups on pyridine ring, and the formation of super-conjugated system between the pyridyl and carbon-nitrogen p-π conjugate groups, the dihedral angles of that in O-CSP1, O-CSP2, O-CSP3 and O-CSP4 (15.24°, 20.32°, 13.73° and 5.37°) are relatively small. However, the bond length between the pyridyl and nitrogen atom are different in O-CSPX. Due to the fluorine atom is a strong electron withdrawing group, the bond length of O-CSP4 is the shortest, while the methyl group is an electron donating group, and the bond length of O-CSP2 is the longest.

From Figure S2, these are the lowest energy forms of O-CMPX-M. The dihedral angles between the pyridyl and carbon-nitrogen p-π conjugate groups in O-CSP1-Cu, O-CSP1-Zn, O-CSP1-Ni, O-CSP2-Cu, O-CSP2-Zn, O-CSP2-Ni, O-CSP3-Cu, O-CSP3-Zn, O-CSP3-Ni, O-CSP4-Cu, O-CSP4-Zn and O-CSP4-Ni (25.68°, 34.75°, 30.14°, 26.74°, 36.12°, 31.06°, 25.39°, 34.67°, 29.22°, 21.74°, 31.15° and 26.04°, respectively) are relatively larger than O-CSPX. The result is due to the influence of the metal ions. The bond length between carbon atom and metal ion (C-M bond length) was also influenced by the substituents on pyridine. The stronger the electronegativity of the substituent group, the lower the electron cloud density of the carbon-nitrogen p-π conjugate groups, leading to the longer C-M bond length and the easier dissociation of the ions. In O-CSPX-M, the Cu^2+^ and Ni^2+^ underwent dsp^2^ hybridization, the Zn^2+^ underwent sp^3^ hybridization, because of the steric hindrance, the nitrogen atoms in the molecule did not form the coordination bond with the metal ions. The charge of the carbon atom in the p-π conjugate group is -0.549e; therefore, we can infer that the carbon atom and the oxygen atoms in the acetate ion became the ligands of the metal ions. This illuminates the molecular configuration of O-CMPX-M, and consistent with our previous research.

Figure S1

Molecular unit configuration of O-carboxymethyl chitosan Schiff bases (O-CSPX)


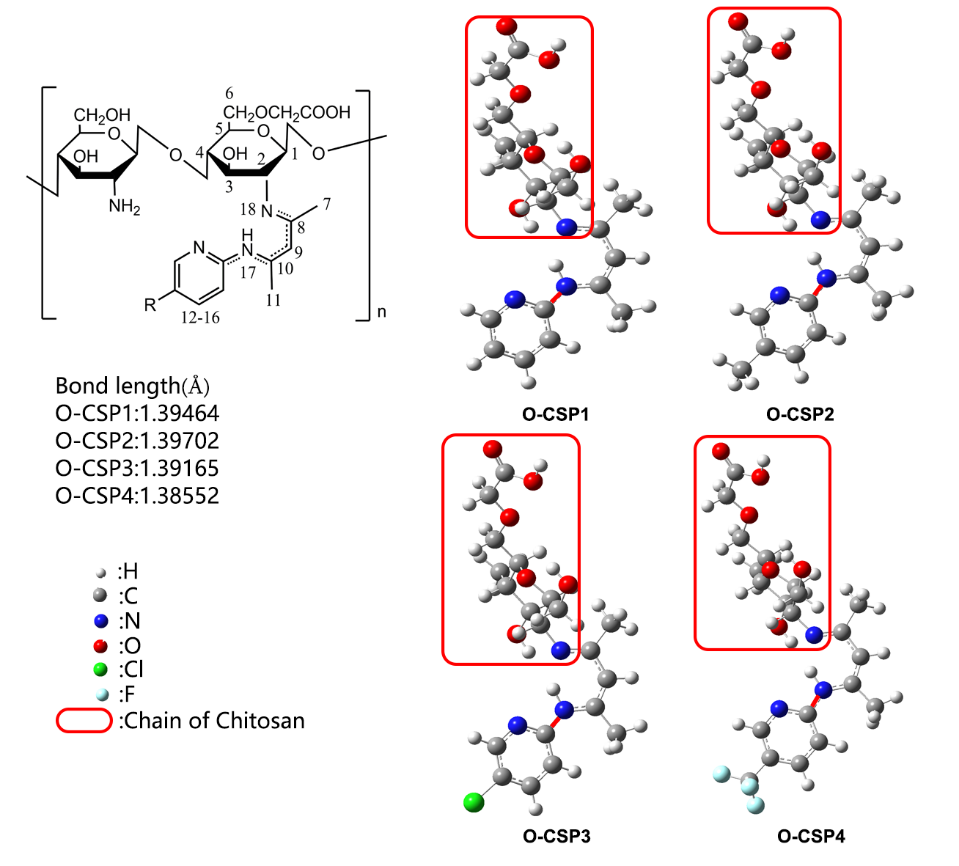


Figure S2

Molecular unit configuration of O-carboxymethyl chitosan metal complexes (O-CSPX-M)


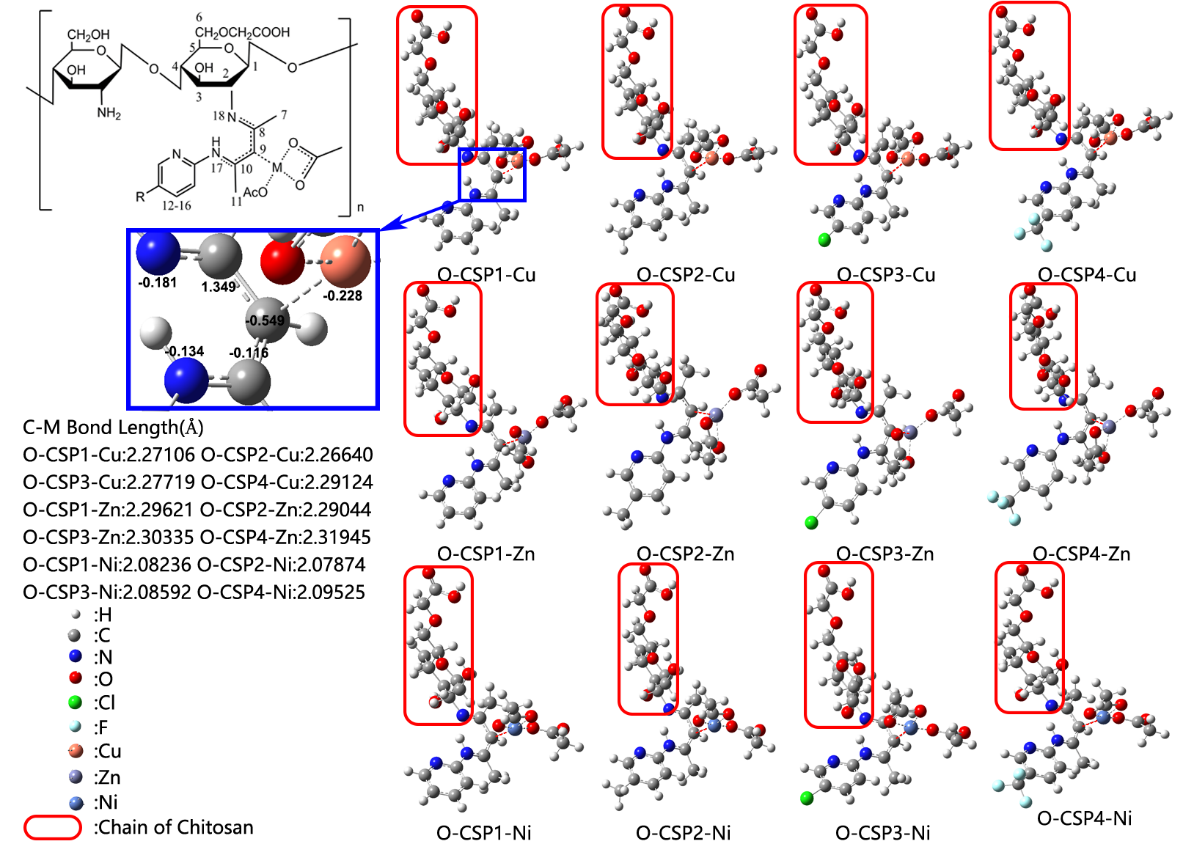


Figure S3

Inhibitory effect of different concentrations of acetic acid aqueous solution on *P. capsici*, *V. alboatrum*, *B. cinerea* and *R. solani*





Figure S4

^1^H NMR spectra of Schiff base ligands (PX)


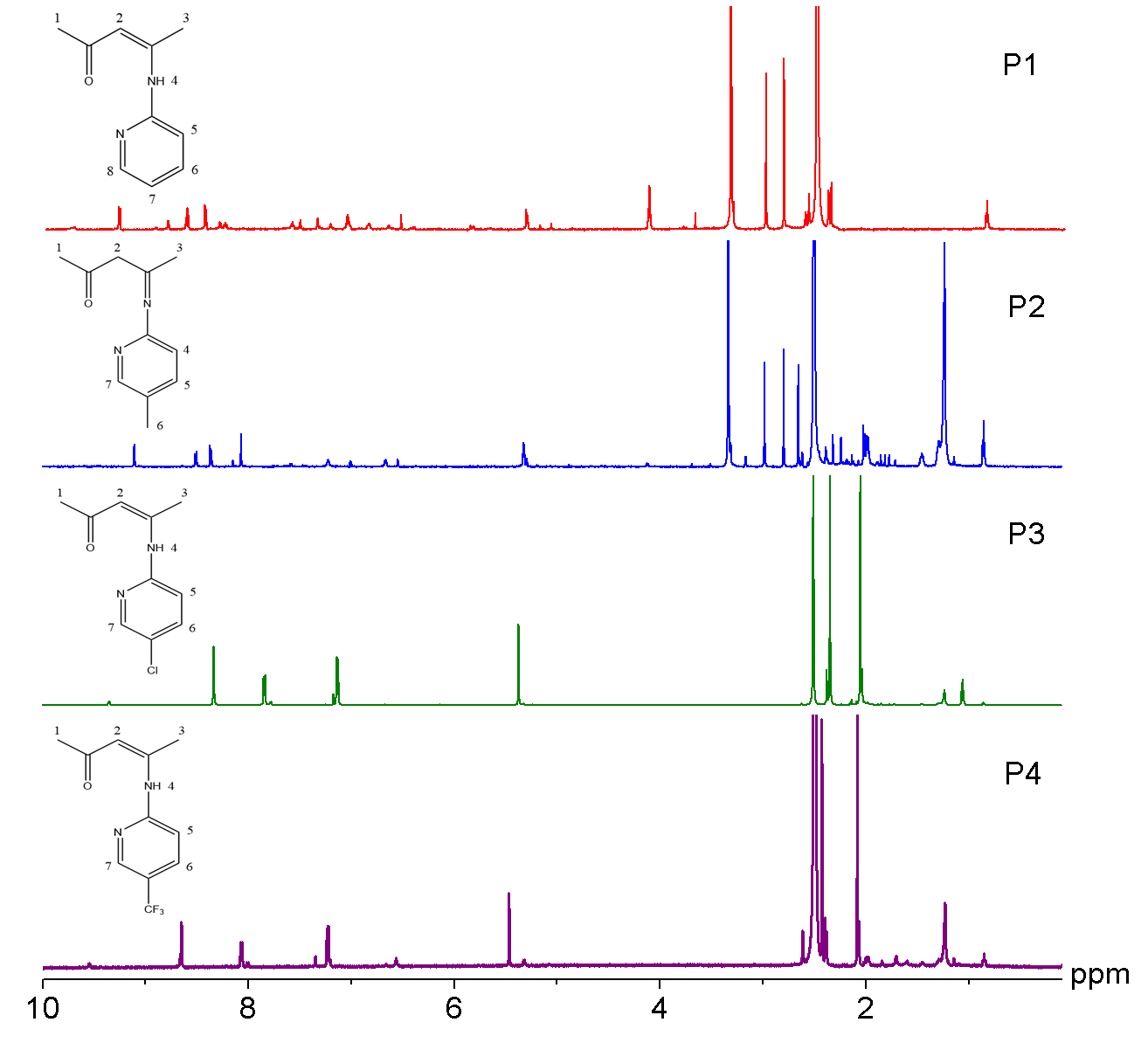


P1: ^1^H NMR, δ: 2.83 (s, 3H, H_1_), 3.00 (s, 3H, H_3_), 5.33 (s, 1H, H_2_), 7.06 (t, 1H, H_7_), 8.45 (d, 1H, H_5_), 8.62 (t, 1H, H_6_), 9.29 (d, 1H, H_8_).

P2: ^1^H NMR, δ: 2.65 (s, 3H, H_6_), 2.80 (s, 3H, H_3_), 2.98 (s, 3H, H_1_), 5.31 (t, 2H, H_2_), 8.37 (d, 1H, H_4_), 8.50 (d, 1H, H_5_), 9.10 (s, 1H, H_7_).

P3: ^1^H NMR, δ: 2.05 (s, 3H, H_3_), 2.35 (s, 3H, H_1_), 5.37 (s, 1H, H_2_), 7.14 (d, 1H, H_5_), 7.84 (d, 1H, H_6_), 8.33 (s, 1H, H_7_).

P4: ^1^H NMR, δ: 2.09 (s, 3H, H_3_), 2.43 (s, 3H, H_1_), 5.47 (s, 1H, H_2_), 7.22 (d, 1H, H_5_), 8.06 (d, 1H, H_6_), 8.65 (s, 1H, H_7_).

Figure S5

Protective and curative activity of O-carboxymethyl chitosan copper complexes (O-CSPX-Cu)


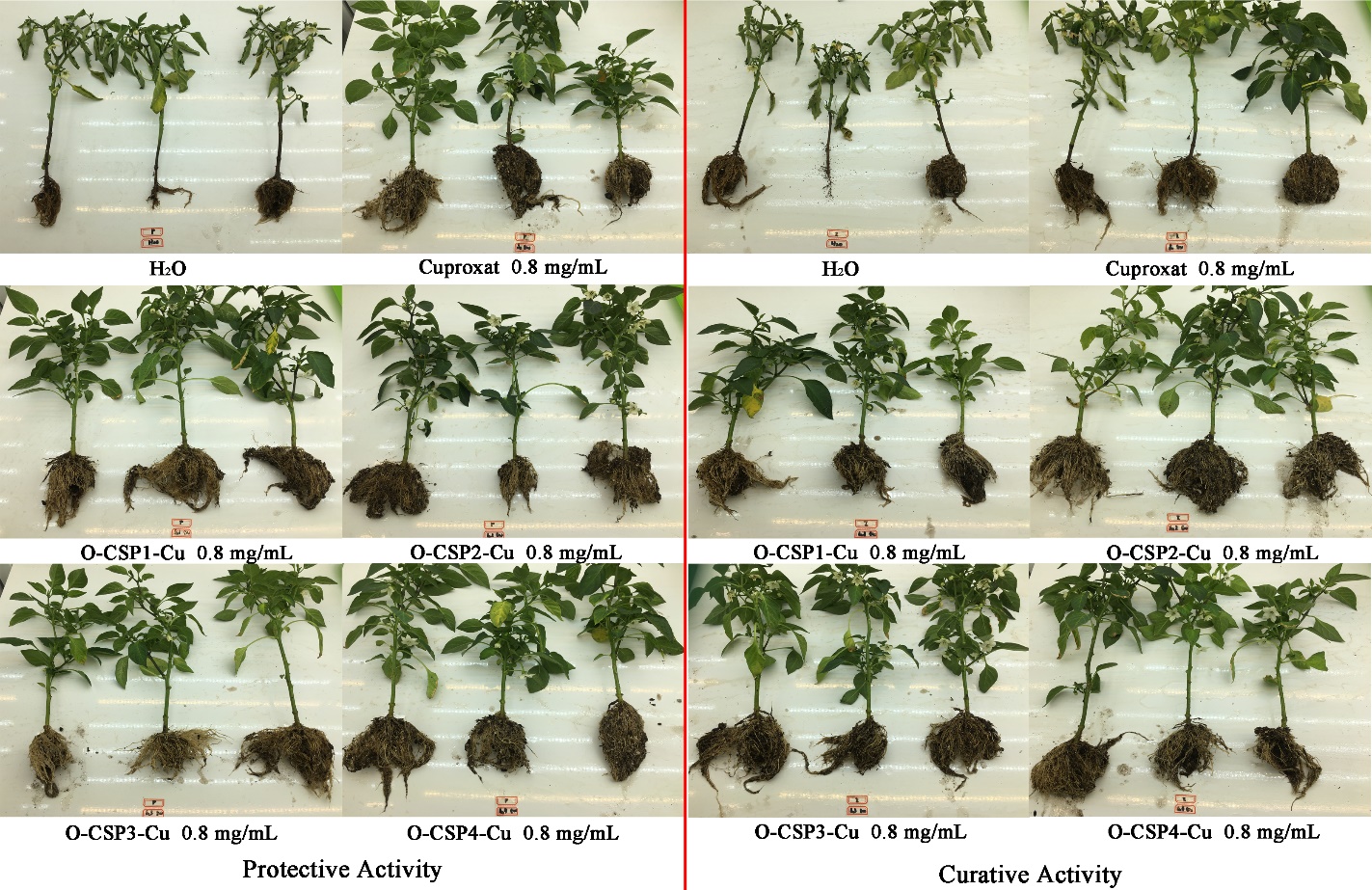


Table S1

Elemental analysis results and degree of O-carboxymethyl chitosan Schiff bases (O-CSPX)

| Compound | Found (%) | | Calculated (%) | | Degree of deacetylation or substitution (%) |
| --- | --- | --- | --- | --- | --- |
|  | N | C | N | C |  |
| CS | 8.44 | 43.39 | 8.69 | 44.72 | 100 |
| O-CSP1-Cu | 3.82 | 28.90 | 7.51 | 47.23 | 71.36 |
| O-CSP2-Cu | 3.96 | 30.99 | 7.33 | 48.17 | 70.08 |
| O-CSP3-Cu | 4.27 | 32.07 | 7.08 | 44.48 | 70.58 |
| O-CSP4-Cu | 4.01 | 31.79 | 6.70 | 44.02 | 76.54 |
| O-CSP1-Zn | 4.66 | 34.77 | 7.50 | 47.14 | 69.88 |
| O-CSP2-Zn | 4.51 | 34.42 | 7.32 | 48.08 | 67.49 |
| O-CSP3-Zn | 4.66 | 34.09 | 7.06 | 44.41 | 67.79 |
| O-CSP4-Zn | 4.43 | 34.39 | 6.69 | 43.95 | 74.19 |
| O-CSP1-Ni | 4.44 | 33.00 | 7.58 | 47.65 | 69.46 |
| O-CSP2- Ni | 4.17 | 31.87 | 7.39 | 48.59 | 67.64 |
| O-CSP3- Ni | 4.46 | 32.53 | 7.14 | 44.86 | 67.48 |
| O-CSP4- Ni | 4.29 | 32.96 | 6.75 | 44.37 | 73.04 |

Table S2

The content for the metals in O-carboxymethyl chitosan Schiff bases (O-CSPX)

| Compound | Metal content (%) | | |
| --- | --- | --- | --- |
|  | Cu | Zn | Ni |
| O-CSP1-Cu | 21.55 |  |  |
| O-CSP2-Cu | 20.52 |  |  |
| O-CSP3-Cu | 15.83 |  |  |
| O-CSP4-Cu | 16.28 |  |  |
| O-CSP1-Zn |  | 13.97 |  |
| O-CSP2-Zn |  | 14.32 |  |
| O-CSP3-Zn |  | 13.83 |  |
| O-CSP4-Zn |  | 13.94 |  |
| O-CSP1-Ni |  |  | 12.20 |
| O-CSP2- Ni |  |  | 13.43 |
| O-CSP3- Ni |  |  | 11.91 |
| O-CSP4- Ni |  |  | 12.72 |
